# Additional Remarks upon the Treatment of Dysentery

**Published:** 1853-02

**Authors:** 


*Additional Remarks upon the Treatment of Dysentery.* By E. F. Starr, M. D., of Rome, Ga.

In a former number, I offered a few remarks upon dysentery, and the use of large doses of opium in its treatment. I now propose to direct attention to a plan of treatment which differs somewhat from that in general use. I do this, because I believe there is some room for improvement, and that the want of success is not to be charged altogether to a fault in the resources of the materia medica. It is a fact worthy of some notice, that, of late years, while medicine has been triumphing in so remarkable a manner over some forms of disease, dysentery has not been shorn of its terrors, and its treatment now is little more successful than it was many years ago. As we have certainly not arrived at a point beyond which we may not advance, we are justifiable in casting around us to see if there be not means as efficient for the relief of other diseases as quinine is for the cure of autumnal fevers. Is there no reliable remedy for dysentery?

If I am told there is a vast weight of authority in favor of the calomel treatment, and that it has been the established practice for scores of years, I appeal to the number of deaths by the disease at the present day. Comparatively but a few years ago, calomel was considered the great remedy for autumnal fevers; yet the physician who knows no better now is far behind the times. I esteem calomel very highly as a remedy; nor is my design so much to wage war against it, as to place it in the back ground, compared with opium, in the treatment of dysentery. I am disposed to "retain the mastodon in harness," and to make it useful by judicious and cautious administration whenever necessary, which will rarely be the case in dysentery. A patient with sound bowels may, perhaps, take a full purgative dose of calomel with impunity, but that one with inflamed intestines can do so with entire safety admits of much doubt. Unfortunately, from the word bile has been derived bilious, and from this, bilious attacks, and bilious fever, and bilious colic, and bilious dysentery, and bilious every thing else to which the term could be applied; and to this prevalence of the *bilious idea* is to be attributed the belief, that to cure these affections there is little more necessity than to purge out the bile and "regulate the secretions," with calomel, of course.

Dysentery is a primary inflammation of the parts ostensibly affected, and it is not to be expected that it can be promptly and certainly cured, without directing attention to the condition of the diseased locality; nor need we hope to obtain success in the use of an irritating and motor-exciting treatment: for an inflamed organ needs rest, and to place it in the most favorable conditon for a restoration to health, it must have rest. The hazard to which a little untimely exertion may subject a patient suffering with acute inflammation of any important organ is well known—how certainly then must undue action in an organ, itself inflamed, produce injurious effects. The value of opium, therefore, and the danger of a different class of agents, must be apparent under this view of the subject.

With regard to the use of cathartics, I would not be misunderstood: they are sometimes useful and necessary, but should, in such cases, be cautiously used, and, in general, be combined with opiates. I think there is often too much anxiety among practitioners upon the subject of scybalæ, and too great a passion for producing discharges of fœcal matter; yet I would not produce the impression that the contents of the bowels are to be left entirely without attention, but, that they are of little importance compared with the *intrinsic features* of the disease. In confirmation of this view, I will quote a fact from Dr. Brandon's article in the March No. of this Journal. In describing some violent cases, which came under his notice, he says, that he "seldom observed fœcal matter in the discharges until a change for the better had occurred." The improvement, then, could not have been produced by the feculent discharges, but these must have been the result of an amelioration of the disease—showing that the disease may be first subdued by opium and other remedies, and the bowels afterward emptied more safely.

In the treatment of dysentery, our object should be to effect, promptly, the cessation of spasm and pain, and the reduction of inflammatory and febrile action. These indications are all under the control of the therapeutic properties of opium, to a greater or less extent; and if these indications and these properties are properly weighed and considered, in connection with each other, the tendency will be toward the establishment of the treatment which I am endeavoring to urge upon the attention of those who may notice these suggestions. The reason why so little reliance has been placed in opium, is, that it has not been given in sufficient quantities—in doses large enough to overcome the force of disease, and to produce its legitimate and peculiar antiphlogistic and antifebrile effects. There is no confidence to be placed in an ordinary or medium dose of opium when the patient is suffering the effects of violent inflammatory action, the tortures of pain, or the depressing adynamic influence of malignant disease. The dose must be proportionate to the emergency of the case. I suggested from two to four grains, but this should not be considered the limit; this quantity is rather the minimum than the maximum—circumstances must determine the precise amount. In dysentery, if the pain, fever, and flux persist, they are sufficient evidence that enough has not been given—six grains are not too much, in such cases. The antiphlogistic virtues of opium seem generally to be imperfectly known or understood, or if known, not appropriated and applied. All agree in admitting its usefulness as an anodyne, as a soother of pain and promoter of sleep, etc.; but who administers it with a view of overcoming fever, or who looks to it principally to subdue some severe forms of inflammation. Yet, what diaphoretic will produce such certain and general opening of the pores and genial moisture of the surface?—what will so equalize the circulation?—what so control the heart and arteries? and what afford such suspension of pain, thereby breaking the *chain* of the morbid actions of inflammation? Fever and inflammation cannot well persist under such circumstances—under the effects of full doses of opium.

To carry out more effectually the suggestion above made, in relation to the indications of treatment, it may be often proper to resort to one efficient bloodletting, in cases where there is much fever and no want of strength. This will render the system more susceptible to the favorable influence of opium, which now, if properly administered, will never fail to mitigate, and seldom to relieve entirely, the sufferings of the patient. When this is done, the use of opium is not to exclude other substances as auxiliaries; such, for instance as calomel or oil, when they are needed, or sugar of lead and other astringents, when, after the subsidence of the inflammatory symptoms, the discharges remain too frequent and watery. These, with fomentations, blisters, enemata of watery solution of opium and starch, &c., may be resorted to; but opium in large doses, given either by the mouth or rectum, in the early stage of the disease, should be the leading remedy and chief reliance.—*Southern Med. and Surg. Journal.*

